# Plant metabolomics: a new strategy and tool for quality evaluation of Chinese medicinal materials

**DOI:** 10.1186/s13020-022-00601-y

**Published:** 2022-04-08

**Authors:** Qi Xiao, Xinlu Mu, Jiushi Liu, Bin Li, Haitao Liu, Bengang Zhang, Peigen Xiao

**Affiliations:** Key Laboratory of Bioactive Substances and Resources Utilization of Chinese Herbal Medicine (Peking Union Medical College), Ministry of Education, Institute of Medicinal Plant Development, Chinese Academy of Medical Sciences, Peking Union Medical College, Beijing, 100193 China

**Keywords:** Chinese medicinal materials, Plant metabolomics, Quality control, Biomarkers, Multivariate statistical analysis

## Abstract

The present quality control method of Chinese medicinal materials (CMM) has obvious deficiency, which cannot be compatible with the multi-target and multi-component characteristics and production process of CMM. Plant metabolomics with a huge impetus to comprehensively characterize the metabolites and clarify the complexity and integrity of CMM, has been widely used in the research of CMM. This article comprehensively reviewed the application of plant metabolomics in the quality control of CMM. It introduced the concept, technique, and application examples, discussed the prospects, limitations, improvements of plant metabolomics. MS and NMR, as important techniques for plant metabolomics, are mainly highlighted in the case references. The purpose of this article is to clarify the advantage of plants metabolomics for promoting the optimization of the CMM quality control system and proposing a system approach to realize the overall quality control of CMM based on plant metabolomics combined with multidisciplinary method.

## Introduction

Chinese medicinal materials (CMM) from plant sources are plants with preventive and therapeutic effects in diseases. China has more than 10,000 kinds of medicinal plants, about 87% of the total number of CMM resources. Different therapeutically active ingredients, from plant sources, have long been used in China. Some CMM with dual-purpose of drug and food, such as peach, jujube and plum, have been recorded as early as 2600 years ago. The Chinese pharmacopoeia (ChP) (2020 edition) has documented 499 categories of CMM from plant sources. In recent years, especially in fighting against COVID-19, CMM has played a significant role, such as forsythia, honeysuckle and ephedra in Lianhua Qingwen capsules (granules) [1, 2]. In so that, CMM attracted a growing amount of attention as the choice of clinical treatment and the source of new drug discovery. A controlled quality evaluation standard is a national strategy for the development of scientific industry, the modernization and internationalization of CMM, as well as the indispensable guarantee of safety and effectiveness in clinical treatment [3, 4].

**Fig. 1:**
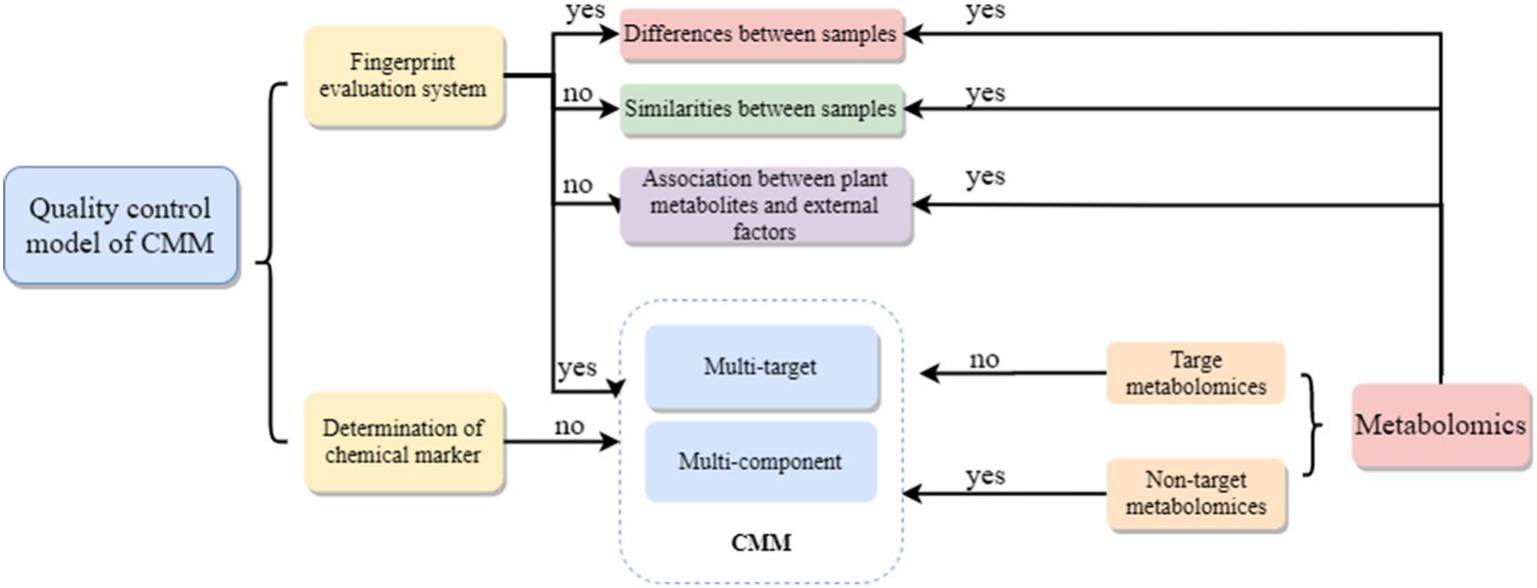
Comparison of existing CMM quality control system and metabolomics methods

The quality of CMM is closely related to their metabolites. CMM biosynthetic secondary metabolites with changeable structure and diverse activities are usually the pharmacodynamic basis of CMM. During the production, these metabolites are prone to interference and produce changes, affecting the quality of CMM [5, 6]. It is difficult to achieve the scientific evaluation of the quality of CMM by current quality evaluation mode which is mainly consisted of determination of chemical markers and the fingerprint evaluation system. The former takes the single component as the evaluation mark, which cannot comprehensively represent the components of CMM [7], while the latter focuses on the similarity between samples and is therefore difficult to evaluate the differences [8, 9] (Fig. 1).

Plant metabolomics is well suited for the quality control of CMM due to it can conduct a comprehensive detection of the metabolites of living organisms. It can be used to qualitatively and quantitatively analyze all small molecular metabolites of plants with different species, genotypes and ecological types, and find the difference between samples [10]. At present, plant metabolomic has been mainly applied to the source identification, authenticity identification, geoherbalism analysis, processing method evaluation and other quality control links of CMM [11, 12]. Mass spectrometry (MS) and nuclear magnetic resonance (NMR) are important analytic techniques, widely used in metabolomics studies. We introduce the principles, the technical composition of plant metabolomics, and collect its application in the quality evaluation of CMM in recent years. Mass spectrometry (MS) and nuclear magnetic resonance (NMR) are important detection techniques of plant metabolomics, their application were highlighted in the paper. Moreover, advantages and disadvantages of current analytical strategies in the innovations of CMM quality were discussed, and a systems strategy to substantially improve the performance of current strategies towards a new-horizon solution to systems-level quality evaluation of CMM was proposed.

## The concept and major technology of plant metabolomics

### The concept of plant metabolomics

Plant Metabolomics, a branch of metabolomics, is designed to study the overall changes in a large number of metabolites in plant samples and then conduct deep data mining and bioinformation analysis. Metabolites, components of defense systems developed by plants in response to pathogen attacks and other environmental stresses, are an important source of many natural pharmacological activity products which are often designated as specific biological activities associated with their biochemical structures [5, 6].

### The main techniques of plant metabolomics

Complete plant metabolomics research process includes experimental design, sample collection, sample processing, sample preparation, detection and analysis, data processing, and metabolic pathway analysis/metabolic network analysis [13]. Analytical techniques and data processing techniques are the two important parts of metabolomics and metabolomics strategy is based on three main techniques: NMR, MS and chromatographic techniques [11, 12]. The common techniques applied to the studies of plant metabolomics are described below. Additionally, MS and NMR are the most advanced detection technology in recent years, with great application potential, and we mainly discuss their advantages and disadvantages.

#### Separation technology

Chromatography is mainly applied to the separation of metabolites GC is mostly used in the study of CMM rich in volatile oils, such as *Cyperi Rhizoma*, *Myristica fragrans Houtt*, and *Rhizoma Chuanxiong* [14–16]. High performance liquid chromatography (HPLC) and ultra performance liquid chromatography (UPLC) are appropriate for the separation and analysis of substances with high boiling points, macromolecules, strong polarity, and poor thermal stability [17–20]. CE achieves a breakthrough in analytical chemistry from microrise to nanorise, making a single cell or even single molecule analysis possible [21, 22]. CE is particularly suitable for the analysis of (highly) polarity and charged metabolites such as amino acids, nucleotides, small organic acids and phosphoric sugars [23–27].

#### Detection technology

The detection techniques of plant metabolomics mainly include UV, IR, MS, NMR. MS is characterised by extreme sensitivity and can provide qualitative and quantitative information according to the molecular weight [28–30]. Electrospray ionization (DESI) can achieve open environmental detection real-time analysis, which is more suitable for the analysis of drugs and endogenous small molecules. Orbitrap analyzer, time-of-flight (TOF) analyzer, and fourier transform ion cyclotron resonance (FTICR) analyzer for high resolution MS, enables the determination of fine structure [31]. The disadvantages of MS include inaccurate identification of unknown compounds, quantitative analysis depending on the reference substance, and the need of purity of the mixture and prior separation. MS is often coupled with a separation technique, such as liquid chromatography (LC), gas chromatography (GC) or capillary electrophoresis (CE) [32].

NMR, especially ^1^H-NMR, ^1^C-NMR and ^31^P-NMR, in plant metabolomics, commonly used in identification of species and growth years, metabolic pathways research, pharmacodynamics study, and methodology evaluation of CMM. NMR shows the absolute advantage in the identification for unknown compounds, which is of value in new compound discovery [33]. The sample preparation of NMR is simple, 1H high resolution magic angle rotation (HRMAS) can even be used for direct sample analysis [34]. In addition, sample detection of NMR is non-destructive and can be associated with downstream detection to achieve high-throughput determination [35]. However, even if new probes such as cryogenically cooled probes and microcoil probes appear, it is difficult to surpass the MS in sensitivity. In recent years, some studies have shown that the small high-temperature superconducting coils and ultra poltechnology can improve NMR sensitivity, but not widely used [36].

In addition, ultraviolet spectrum (UV) as a fundamental analysis technique has been widely used in the research of CMM [37, 38]. In recent years, infrared (IR) was applied to the study of enzyme activity, grease and fat, species difference, harvesting time, and source of CMM [39–43].

#### Data processing techniques

**Fig. 2:**
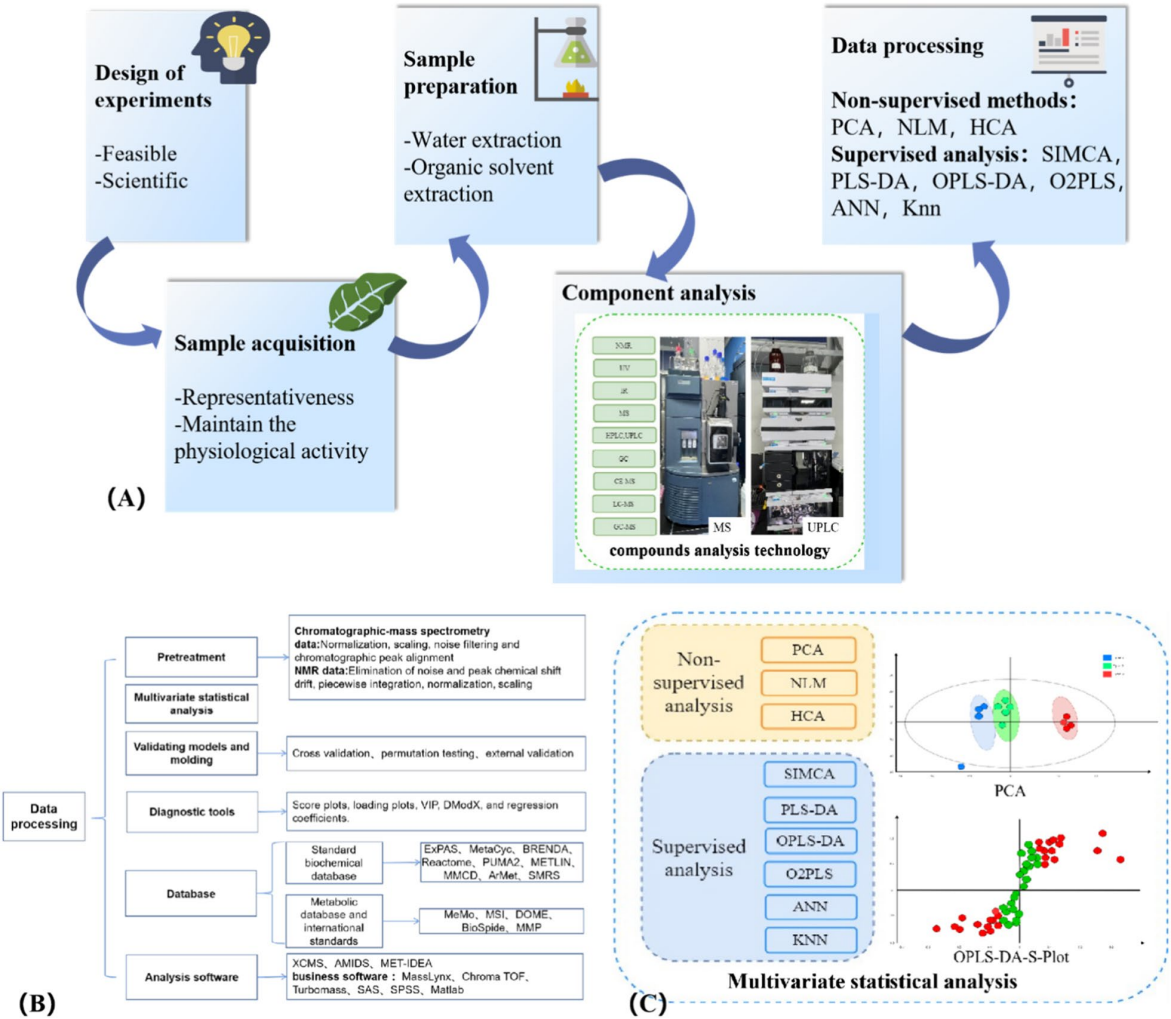
Process, technology and methods of plant metabolomics: **A** The process of plant metabolomics. **B** Contents included in the data processing [111–113]. **C** The classification of multivariate statistical methods and figure of PCA and OPLS-DA [114–118]

The pattern recognition technology is mainly for data processing, including unsupervised analysis and supervised analysis [44–47]. Principal components analysis (PCA) can clearly demonstrates repeatability within data group and differences between data groups [49], describing the data in a minimum dimensions but guarantee reproducibility. Partial least squares discriminative analysis (PLS-DA) and orthogonal projections to latent structures (OPLS-DA) reduce the data dimensionality and analyse them with a regression model [50]. Compared to PCA, PLS-DA/OPLS-DA establishes the model between metabolic expression and grouping relations, which better obtains intergroup difference information and predicts the grouping of samples [51]. In a practical application, several statistical methods are often combined according to the data characteristics and research requirement. Necessarily, these detection and analysis techniques depend on existing software and databases. (Fig. 2).

## The application of plant metabolomics in quality evaluation of Chinese medicinal materials

**Fig. 3:**
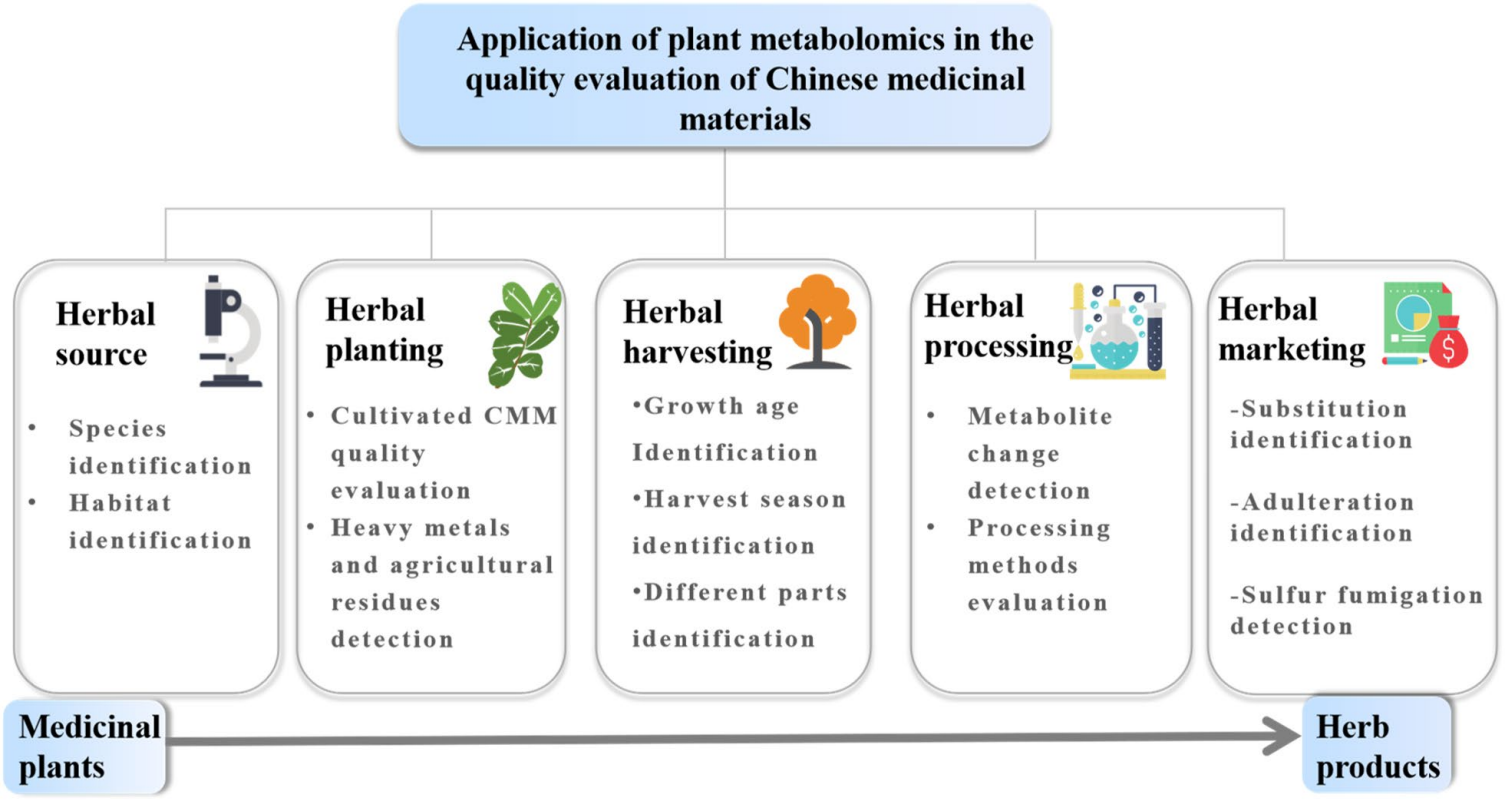
The application of plant metabolomics in the quality control of CMM

Unlike chemosynthetic drugs, CMM is a complex system composed of multiple chemical components. It is characterized by diverse chemical compositions, diverse structures and huge content differences [51, 52]. In addition, the species and contents of chemical components are susceptible to be influenced by factors such as germplasm resources, growth conditions, cultivation and processing methods, etc. [53–55]. MS-and NMR-based metabolomic techniques can be used to monitor key indicators of the production link of CMM by analyzing changes in the type and content of metabolites. Below we mainly discuss the application of MS-and MNR-based metabolomics techniques in five production links of CMM: sources, planting, harvesting, processing and market sales (Fig. 3).

### Herbal source

The types and contents of the active metabolites in CMM are highly variable depending on the species, parts of the plant, cultivated geographic region, and planting period involved.

#### Species identification

Species genetically determine the difference in types and contents of plant metabolites, leading to differences in clinical effects. The current ChP stipulates that the original plant of licorice herbs is *Glycyrrhiza Uralensis, Glycyrrhiza inflata* or *Glycyrrhiza glabra.* The chemical composition and efficacy of the three kinds of licorice herbs differ significantly, but the mixing use of same generic plants often occurs frequently. Fukuda et al. used direct analysis in real time (DART)-MS to detect the metabolites of several *licorice* species, the peak at m/z 339 was mainly derived from [M + H]^+^ of licochalcone A, which is present in *Glycyrrhiza inflata* and is hardly detected in *Glycyrrhiza glabra* and *Glycyrrhiza Uralensis*. Therefore, licochalcone A can serve as a candidate biomarker to distinguish these three *licorice* species, which has great implications for ensuring the clinical efficacy of licorice [56]. In one study, in vitro activity experiments and NMR based metabolomic were combined to comprehensively measure the activity differences of *Artemisia afra* and *Artemisia annua,* two antimalarial drugs come from Africa and China. The antiplasmodial activity of *Artemisia afra* and *Artemisia annua* extracts were tested for activity against *Plasmodiam falciparum* 3D7, and the activity was only found in polar extracts of *Artemisia annua*. 1D- and 2D-NMR spectroscopy identified 24 semi-polar components including three novel phenylpropane for this species: caffeic acid, chlorogenic acid and 3,5-dicaffeoyl quinic acid, which can be seen as the biomarkers of this species. Combined with the in vitro experiments, metabolomics allows for a more comprehensive evaluation of the quality of CMM based on the compounds and activity [57]. The combination of metabolomics and molecular techniques shows the unique advantages of species identification. GC and TOF–MS combined with DNA molecular markers was applied to distinguish *Astragalus mongolica* and *Astragalus membranaceus.* In a result, three markers and eight metabolites were identified as candidate DNA and metabolic markers [58]. 1H-NMR combined with DNA barcoding technique ssuccessfully demonstrated that different genetic variation and chemical constituents existed among 3 different Seabuckthorns, *Hippopahe gyantsensis.*, *Hippopahe Neurocarpa* and *Hippopahe tibetana* [59]. The combination of molecular techniques and MS and/or NMR based metabolomics reveals the nature of metabolite changes at the genetic level, which is conducive to the exploration of metabolic rules and functional compound synthesis using molecular technology.

#### Habitat identification

The variety, quantity and quality of CMM resources are restricted by the natural conditions of the cultivated geographic region. The habitat of ginseng has an important impact on the type and number of ginsenosides which are often used as biomarkers to evaluate the quality of *Panax ginseng*. Song et al. successfully identified the difference between Korean white ginseng (KWG) and Chinese white ginseng (CWG) using UPLC-Q-TOF–MS and OPLS-DA. Ginsenoside Rf and notoginsenoside R3 isomer in KWG can be used as biomarkers to make a distinction with the CWG which is rich in ginsenoside Ro and chikusetsusaponin IVa [60]. Dae et al. distinguished *Panax ginseng* grown in Korea, China and Japan by using rapid resolution liquid chromatography (RRLC)-QTOF/MS combined with PCA and PLS-DA. In the PCA score plot, ginseng samples from the three regions were significantly separated, revealing the obvious effect of the planting region on the metabolites [61]. The LC–MS based metabolomics technology has been widely used in the identification of *Panax ginseng* from different habitat, providing a powerful method to ensure the quality of valuable CMM. *Astragalus membranaceus* and *Paeonia albiflora* with different origins in Korea and China were analysed by 1H NMR and inductively coupled plasma atomic emission spectro/inductively coupled plasma-mass spectrometry (ICP-AES/ICP-MS). The results indicated that the complementary multielement and metabolomic data were more suitable for determining the geographical origin than the use of each individual data set alone [62]. HPLC coupled with Q-TOF–MS was used to analyze metabolites from dandelions (*Taraxacum mongolicum*) from four different geographical regions in China, namely Gansu, Henan, Shanxi, and Jiangsu. OPLS-DAD was applied to analysis metabolite and MBRoleto was used to identify potential metabolic pathways. This study found that the differential metabolites of dandelion in these four regions were mostly phenolic, and the biosynthesis of phenylpropanoids and flavonoids were main metabolic pathways involved. Environmental stress generates an enhancement of these metabolic pathways, causing elevated metabolite concentrations, so the samples from Gansu has higher quality relatly. This study not only analyze the metabolite differences between samples, but also conducted the exploration of metabolic pathways to speculate on the association between climate factors and metabolites, which can be used to guide production [63].

### Herbal planting

The higher market demand and the sharp decrease of wild herbal resources encourage the development of herbal planting, which can ease the current contradiction between medicinal resources supply and demand. The problems such as evaluating and comparing the quality of the cultivated and wild, residues of pesticide and herbicides, and excessive use of chemical fertilizers have aroused widespread concern.

#### Cultivated CMM quality evaluation

Although artificially cultivated CMM are booming, cultivated products and wild products often differ in the content of active compounds, which may affect the clinical efficacy of CMM. Agarwood, an aromatic medicinal materials mainly formed from species of *Aquilaria*, is mainly rich in 2-(2-phenylethyl) chromones. Based on characterization of the 2-(2-phenylethyl) chromones via ultra-performance liquid chromatography coupled with electrospray ionization mass spectrometry (UPLC-ESI-QTOF-MS), 14 biomarkers were found from wild and cultivated agarwood. Unfortunately, some chromone isomers were difficult to distinguish clearly due to the most of the compounds have the same molecular formula and similar fragment ions, which requires more sensitive detection methods [64]. Cultivated *Panax quinquefolius*, a very common analogue of wild products, is hard to distinguish in the traditional way due to complex ingredients. 1H NMR-PCA was applied to study 37 batches of sample from 15 pharmacy stores over 5 provinces in China, which built a model which excises the most abundant metabolites of *Panax quinquefolius* extract from the NMR spectra before PCA, and correlated the differences between *Panax quinquefolius* types to the less abundant metabolites. It is the first time for the 1H NMR used in *Panax quinquefolius* and large sample size studies, which provided a new method for other CMM study [65]. According to the growth environment and the cultivation method, ginseng can be divided into three types: cultivated ginseng (CG), mountain-wild ginseng (MWG), and mountain-cultivated ginseng (MCG). The ChP also classified ginseng into CG and MCG groups, and there was no a viable way to distinguish them before. Xu et al. identified 40 ginsenosides in MCG and CG using a plant metabolomics method consisted of UPLC-QTOF-MS/MS and multivariate statistical methods. This is the first observed chemical composition differences between MCG and CG, providing theoretical guidance for the subsequent cultivation of high-quality ginseng [66]. The root of wild *Salvia miltiorrhiza* is red owing to the periderm is rich in tanshinones while the root in cultivated fields showed orange. In a study, metabolome and transcriptome analyses were integrated to analyze the differences of metabolites between two Danshen (*Salvia miltiorrhiza*) in different colors. UPLC/Q-TOf–MS based metabolomic detected 40 lipophilic components and 7 of them including tanshinone IIA and tanshinone I were obviously decreased in the orange one. The results of transcriptome analysis indicated that decreases in the content of dehydrogenated furanring tanshinones such as tanshinone IIA resulted in phenotypic change and quality degradation of Danshen. These changes may be related to environmental deterioration and biological stress. The combination of the metabolome and transcriptome can excavate the factors affecting the quality from the source while conducting the quality evaluation of CMM, which has the guiding significance of the actual production [67].

#### Heavy metals and agricultural residues detection

Heavy metals and agricultural residues are the safety control index of cultivation and the hotspot of quality control of CMM. The accumulation of secondary metabolites in many CMM is related to external environmental stress, such as heavy metals, drought, waterlogging. The research and analysis of stress resistance of CMM in extreme environments are the research trend of future CMM cultivation, which is the research basis for improving the quality of cultivated products. Luo et al. used GC–MS technique, combined with PCA and OPLS-DA, to explore metabolite differences in *Sebum Alfredii* root exudates in different environments. The result displayed that 12 compounds as biomarkers to distinguish different treatment conditions, indicating that *Sedum Alfredii* could tolerate super-rich heavy metal cadmium by regulating their secretion. With the deterioration of the ecological environment, the research on tolerating extreme environmental CMM will inevitably become a hotspot, and these studies will bring the cultivation of CMM to a more promising direction [68]. Moreover, the use of pesticides, including herbicides, fungicides, insecticides, and plant growth regulators, could also affect the secondary metabolism of CMM. UPLC/Q-Orbitrap-Full MS scan method combined with multivariate statistical analysis were employed to evaluate effect of two insecticides, imidacloprid and compound flonicamid and acetamiprid, on the overall composition of *Lonicerae Japonicae Flos* (LJF). The experiment obtained and characterized 29 metabolic markers including iridoids and carried out relative quantitative assay to monitor their changes at different times of flowering developments. These decreased iridoids should be considered as important markers in the holistic quality assessment of LJF. This is the first time to study the impact of pesticides on metabolites of LJF, which provides a basis of pesticide indicators for the quality control of bulk cultivated CMM [69]. The use of pesticides is a necessary link for the mass production of CMM. With the application of plant metabolomics technology, we can find pesticides friendly to plant growth or with less influence, which will promote quality improvement of CMM cultivation (Table 1).

**Table 1:** Application of plant metabolomics in the quality evaluation of CMM

Application	Species	Data processing technique	Analytical techniques	Number of metabolites	Biomarker	Effect	Refs.
Herbal source							
Species identification	*Glycyrrhiza Uralensis, Glycyrrhiza inflata or Glycyrrhiza glabra*	/	DART (Direct Analysis in Real Time)-MS	1	Licochalcone A (LA)	Pain relief, antitussive, anti-inflammatory, anti-allergic, enhanced immunity	[56]
	*Artemisia afra and Artemisia annua*	PCA	1D- and 2D-NMR	24	24 semi-polar components	Anti-malaria, anti-inflammatory, immunomodulatory	[57]
	*Astragalus membranaceus and Paeonia albiflora*	KNN, LDA, SVM, PLS-DA	1H NMR and ICP-AES/ICP-MS	8	4-aminobutyrate, acetate, alanine, arginine, asparagine, benzoate, choline, citrate, glucose, etc	Regulation of immunity, suppression of inflammatory response, anti-oxidation, anti-tumor, anti-infection	[58]
	*Hippopahe gyantsensis., Hippopahe Neurocarpa and Hippopahe tibetana*	PCA	1H-NMR	32	Flavonoids, amino acids, organic acids, and fatty acids	Regulation of metabolism, anti-tumor	[59]
Habitat identification	*Panax ginseng*	OPLS-DA	UPLC-Q-TOF–MS	23	Ginsenoside Rf, notoginsenoside R3 isomer, ginsenoside Ro and chikusetsusaponin Iva	Enhancing learning and memory, strong heart, antishock, anti-myocardial ischemia, enhanced immune function	[60]
	*Panax ginseng*	PCA and PLS-DA	RRLC-QTOF/MS	2	Ginsenosides		[61]
	*Astragalus membranaceus and Paeonia albiflora*	LDA, KNN, SVM, PLS-DA	1H NMR and ICP-AES/ICP-MS	7	Calycosin, formononetin, linoleic acid, etc	Regulation of immunity, suppression of inflammatory response, anti-oxidation, anti-tumor, anti-infection	[62]
	*Taraxacum mongolicum*	PCA, OPLS-DA,	HPLC-Q-TOF–MS	54	Guanosine 50 -monophosphate, phosphoric acid, gallic acid, catechol, citraconic acid, cis-aconitate,, etc	diuresis, anti-inflammatory, and promote digestion	[63]
Herbal planting							
Cultivated CMM quality evaluation	Ggarwood	PCA, OPLS-DA	UPLC-ESI-QTOF-MS	1	2-(2-phenylethyl) chromones	Antioxidant and bacteriostatic, analgesic and sedative, anti-inflammatory, anti-cancer	[64]
	*Panax quinquefolius*	PCA	NMR	6	Sucrose, glucose, arginine, choline, and 2-oxoglutarate and malate	Enhancing learning and memory, strong heart, antishock, anti-myocardial ischemia, enhanced immune function,etc	[65]
	*Panax ginseng*	PCA, PLS	UPLC-ESI-QTOF-MS	40	Ginsenosides		[66]
	*Salvia miltiorrhiza*	PCA, OPLS-DA	UPLC-Q-TOF/MS	17	Alkaloids	Anti-tumor, anti-atherosclerosis, anti-neuroinflammation, inhibiting myocardial hypertrophy	[67]
Heavy metals and agricultural residues detection	*Sedum Alfredii*	PCA, OPLS-DA	GC–MS	12	/	/	[68]
	*Lonicerae Japonicae Flos*	PCA.OPLS-DA	UPLC/Q-Orbitrap-Full MS scan	29	Chlorogenic acids, iridoids and organic acid-glucosides	Anti-virus, anti-lipids, anti-tumor factors, anti-bacterial, anti-allinflammatory	[69]
Herbal harvesting							
Growth age Identification	*Panax ginseng*	PCA	UPLC-QTOF/MS	30	/	Prevent aging, eliminate fatigue, improve memory, anti—aging, anti—tumor	[70]
	*Panax ginseng*	/	HPLC-MRM/MSA	301	ginsenosides	Enhancing learning and memory, strong heart, antishock, anti-myocardial ischemia, enhanced immune function, etc	[32]
	*Polygala tenuifolia Willd*	PCA	UPLC-QTOF-MS	26	Tenufolia, fructose, sucrose, choline, glycine, raffinose, onjisaponin Fg, polygalasaponin XXVIII, etc	Enhance Intelligence, sedative-hypnotic, expelling phlegm and arresting coughing, anti—inflammatory, neuro protection, etc	[71]
Harvest season identification	*Panax ginseng*	OPLS-DA	1H- NMR	24	Ginsenosides, arginine, sterols, fatty acids, and uracil diphosphate glucose–sugars	Enhancing learning and memory, strong hear, antishock, anti-myocardial ischemia, enhanced immune function, etc	[72]
	*Piper Sarmentosum*	PCA	FTIR	/	/	Anti-amoebic, antibacterial, anti-neoplastic, neuromuscular blocking, hypoglycemic, antioxidant,etc	[73]
	*Pericarpium Citri Reticulatae (PCR) and Pericarpium Citri Reticulatae Viride (PCRV)*	PCA	HPLC, HELP	16	Naringin, hesperidin, nobiletin, tangeretin	Anti-inflammatory, anti-oxidation, regulating metabolism, neuroprotection, anti-tumor	[74]
Different parts identification	*Cistanche deserticola*	PLS	UPLC-PDA-Q/TOF–MS	6	Echinacoside, cistanoside A and 2 ' -acetylacteoside	Neuroprotection, immunomodulation, anti-aging, anti-osteoporosis, protecting liver and protecting liver	[75]
	*Ginseng*	PCA, OPLS-DA	UHPLC-QTOF/MS	81	Rutinum, 20(R)-Notoginsenoside R2, Ginsenoside F3, etc	Enhancing learning and memory, strong heart, antishock, anti-myocardial ischemia, enhanced immune function, etc	[76]
	*Gentiana crasicaulis*	PCA, OPLS-DA	UPLC-ESI-HRMSn	36	Hydroxycinnamic acid amides, trans-N-caffeoyl phenethylamine	Anti-inflammatory, anti-allergy, acesodyne	[77]
	*Mulberry*	PCA, SIMCA	1HNMR	24	/	Anti-inflammatory, anti-oxidation, anti-tumor, hypoglycemic, anti-hyperlipidemia, liver protection	[78]
	*Clausena lansium* (Lour.) Skeels	PCA, OPLS-DA	UPLC-Q-Orbitrap-MS	364	β-Alanine, 4-Pyridoxic acid, Phosphorylcholine, N-Acetylglutamic acid, N-Methyltyrosine, etc	Promote digestion, anti-influenza, relieve pain	[79]
Herbal processing							
Metabolite change detection	*Astragali Radix*	OPLS-DA, PCA	UPLC-QTOF-MS	15	Calycosin-7-O-β-Dglucopyranoside, Calycosin, formononetin, (6αR, 11αR)- 10- Hydroxy-3, etc	Anti-tumor, protection of cardio-cerebrovascular, improvement of immune function, etc	[80]
	*Radix Polygala*	/	UPLC-QTOF-MS\NMR	29	3,6′-disinapoylsucrose ester, amino acids,organic acid, and carbohydrates, total saponins	Enhance Intelligence, sedative-hypnotic, expelling phlegm and arresting coughing, anti—inflammatory, neuro protection, etc	[81]
	*Panax notoginseng*	/	1H-NMR/ UPLC	29	Ginsenoside Rf, 20(S)‐pseudoginsenoside F11, malonyl gisenoside Rb1, etc	Anti-cancer, lipid-lowering, neuroprotection, endothelial cell protection, bone repair, anti-fibrosis	[82]
	*Rheum palmatum*	PCA, OPLS-DA	UPLC/Q-TOF–MS	2	Emodin-8-O-glucoside and gallic acid-3-O-glucoside	Improve the digestive system, regulate the blood system, promote metabolism, affect the nervous system, etc	[83]
	*Aconitum carmichaelii Debx*	PLS-DA, PCA	RPLC-Q-TOF/MS	22	Dehydrated Benzoylmesaconine, Dehydrated Benzoylaconine, 10-OH-Benzoylaconine, etc	Anti-inflammatory, relieve pain, anti-tumor, cardiovascular protection	[84]
	*Euphorbia pekinensis Root*	PCA	UPLC-MS	7	3,3'-di-O-methylellagic acid-4'-O-β-D-xylopyranoside,3,3'-di-O-methyl ellagic acid, etc	Reversal of P-glycopprotein-mediated multidrug resistance, cytotoxicity, lipase inhibitory activity, etc	[85]
Processing methods evaluation	*Panax notoginseng*	PLS-DA, PCA	UHPLC/TOF MS	40	Ginsenoside Ra3/isomer, gypenoside XVII, quinquenoside R1, ginsenoside Ra7, notoginsenoside Fe, etc	Anti-cancer, lipid-lowering, neuroprotection, endothelial cell protection, etc	[86]
	*Ginseng*	ANOVA	HPLC	10	Ginsenosides	Enhancing learning and memory, strong heart, antishock, anti-myocardial ischemia, etc	[87]
	*Notopterygium franchetii*	PCA, HCA	UHPLC-QTOF-MS–MS	30	Nodakenin, psoralen, bergapten, notopterol, imperatorin, and isoimperatorin	Anti-inflammatory, anti-oxidation, anti-arrhythmic, anti-bacterial, etc	[88]
	*Salvia miltiorrhiza*	/	NMR and LC-DAD-MS	81	sugars, carboxylic acids and amino acids, polyphenolic acids and diterpenoids, etc	Anti-tumor, anti-neuroinflammation, inhibiting myocardial hypertrophy	[89]
	*Astragalus root*	PCA	H-1 NMR /UPLC-MS	19	acetate, alanine, arginine, caprate, fumarate, glutamate, valine, and some xylem-related compounds	Anti-tumor, protection of cardio-cerebrovascular, improvement of immune function, etc	[90]
Herbal marketing							
	*Hydrastis canadensis(goldenseal) canadensis*	PCA	MS	12	Berberine, hydrastine, canadine, palmatine, coptisine, dihydrocoptisine	Antimicrobial, anti-inflammatory, hypolipidemic, hypoglycemic, antioxidant, neuroprotective, etc	[91]
	*Panax ginseng*	OPLS-DA	1H NMR	15	/	Enhancing learning and memory, enhanced immune function, strong heart, antishock, etc	[92]
	*Panax ginseng*	OPLS-DA, PCA	UPLC-QTOF-MS/MS	43	ginsenoside Rg3, a nitrogen-containing component, ginsenoside 20(R)-Rh, etc	Enhancing learning and memory, strong heart, antishock, anti-myocardial ischemia, etc	[93]
	*Trichosanthis Radix*	OPLS-DA, PCA	UPLC-ESI-QTOF-MS/MS, UPLC-ESI-QTRAP-MS/MS	25	5 sulfur-containing components, 20 non-sulfur marker metabolites	Anti virus, anti—tumor, odinopoeia	[94]
	*Rhodiola*	PCA	NMR, HPTLC	/	salidroside	Lowering blood lipids and blood sugar, improve physical, mental, memory, attention and immunity, anti-aging, etc	[95]
	*Rhodiola*	OPLS-DA	HPLC–UV, NMR	5	Coumarin, Crenulatin, rosavins		[96]
	*Angelica acutiloba*	PCA	GC-TOF–MS	22	Sugars, fructose and glucose, phosphoric acid, proline, malic acid and citric acid	Improving anemia, protecting liver, immunomodulation, anti-tumor, anti-inflammatory	[97]

### Herbal harvesting

The growth age and harvest season directly affect the quality, yield and harvest rate of CMM. Moreover, the types and content of active metabolites in different parts of CMM are different, which determines the clinical selection.

#### Growth age identification

Growth age has a significant impact on the contents of metabolites in plants. Ginseng is easily affected by differences in growth years, and 4, 5 and 6 years old Ginseng is in high demand in the market due to have higher levels of active substances. UPLC-Q-TOF–MS combined with PCA and HCA methods was developed to evaluate various ginsenosides in *Panax ginseng* roots between 1 and 6 years old. The results show that 30 ginsenosides profiling was carried out and 14 ginsenosides in P. *ginseng* roots cultivated for 4, 5, and 6 years can be clearly identified [70]. MALDI-MSI is a powerful tool to localize the components distribution and its cross sections showed the distribution of ginsenosides from *P. ginseng* roots cultivated for 4, 5, and 6 years, which provides the indicator of growth age for the quality control of ginseng [32]. The active compounds of *Polygala tenufolia* is apt to be affected by its growth age, and the market generally considers *Polygala tenufolia* aged 2–3 years of better quality. In Chp, Tenuifolin, Polygalaxanthone III, (3-sinapoyl) fructofuranosyl-(6-sinapoyl) glucopyranoside were measured as the detection index for quality of *Polygala tenufolia.* UPLC-QTOF-MS and NMR based metabolomic technology has found that 1 year-old *Polygala tenufolia* was significantly different from those of 2 and 3 years old due to differences between primary and secondary metabolites, and a portion of those index compounds will decrease accordingly as age grows. This result shows that those primary or secondary metabolites, with marked changes, are perhaps more appropriate quality markers [71]. Growth age is an important problem in CMM, which determines its quality. The UPLC-Q-TOF–MS and NMR methods were applied to the study of growth age of CMM, which effectively solves the problem of distinguishing different years.

#### Harvest season identification

Harvesting in different seasons affects the quality of CMM, because of the formation and enrichment of compounds are affected by seasonal factors. Lee et al. used 1H NMR metabolomics to explore the relationship between ginseng metabolites and climate. The results show that the growing season from March to October is of great importance for the synthesis of ginseng metabolites. Especially, it has been shown that March to May may be the optimal synthesis time for ginsenosides, arginine, steroids, fatty acids and uracil diphosphate glucose-sugar, a process accompanied by accelerating sucrose catabolism that may be associated with climate change, such as sunshine time and rainfall [72]. One study was designed to investigate the appropriate harvest time of *Piper Sarmentosum fruits* to maintain consistency of efficacy and batch reproducibility. Plant metabolomics was applied to detect, quantify and catalogue the time related metabolic processes of the samples. The Fourier transform infrared spectroscopy (FTIR) spectral data were analyzed by stoichiometry and PCA, and identification, classification and differentiation (ICD) evaluation were obtained using PerkinElmer software. These results indicated that the metabolites in the samples varied in all the batches. FTIR fingerprint profiles of the samples in combination with chemometrics is an effective tool. It is concluded from the results of this study that plant metabolomics in combination with chemometrics is an effective tool of fast and easy assessment of similarity of different samples and may be applied as an analytical tool in quality evaluation [73]. *Pericarpium Citri Reticulatae* (PCR) and *Pericarpium Citri Reticulatae Viride* (PCRV)*,* two different maturation stages of the same plant source, have different clinical effects. Based the HPLC fingerprints, heuristic evolving latent projection (HELP) method was applied to obtain two-dimensional data and chemometrics. The growth footprint of Tangerine peels was given by PCA score chart, which show that July may be the best harvest time for PCRV, and November and December are better for PCR [74].

#### Different parts identification

Due to the differences in the distribution of the active compounds, different plant parts have been regarded as different drugs. The Chp also makes detailed requirements for the medicinal parts of medicinal plants. The succulent stem is the medicinal site of *Cistanche deserticola*, and few studies have focused on other parts. In one study, an UPLC-PA-QTOF-MS based metabolomic method was applied to identify the chemical constituents and screen the antioxidant activity profiles of 6 different parts from cultivated *Cistanche deserticola*. An obvious difference was observed between the chemical profiles and content distribution of phenylethanoid glycosides (PhGs). The results showed a significant decreasing trend from the bottom to the top of cultivated *Cistanche deserticola* and the highest content in the stems. This combination of metabolomics and antioxidant activity detection promotes the link between composition and activity, which will characterize the quality of CMM by a more comprehensive way and provide a guarantee for clinical efficacy [75]. Chang et al. utilized UHPLC-QTOF/MS combined with PCA and OPLS-DA analysis methods to identify different parts of ginseng, showing significant differences in chemical composition. A total of 81 major metabolites were detected from 4 different sites of the *ginseng*, and 70 metabolites was finally identified or preliminarily inferred. The plant metabolomics analysis of different parts of ginseng is beneficial to clarify the differences in pharmacodynamic substances of different parts of ginseng for treating different diseases and this method is fast, accurate, and reliable [76]. UPLC-ESI-HRMSn based metabolomic identified 36 biomarkers from different parts of *Gentiana crasicaulis*. The result shows that iridoids are mainly enriched in the root, while flavonoids and triterpenes were mainly concentrated in the aerial part. Through the detection of different compounds, different plant sites can be clearly distinguished [77]. Additionally, ^1^HNMR combined with PCA and SIMCA was successfully used to identify the differences on compounds from 6 sites of *mulberry leaf* [78]. In recent years, a chemical profiling of seven parts of *Clausena lansium* was built by a non‐targeted UPLC‐Q‐Orbitrap‐MS metabolomic method. 364 metabolites including 62 potential biomarkers were selected by the multivariate statistical analysis. Heat map and KEGG annotation showed a significant enrichment of the “Flavone and flavonol synthesis” and “Isoquinoline alkaloid biosynthesis” pathway. This investigation could provide a foundation for the isolation and identification of new constituents from *Clausena lansium* and further clarified the biosynthetic pathway of differential metabolites among various tissues of *Clausena lansium*, which can be used for reference by other studies of CMM [79].

### Herbal processing

Processing of drugs necessarily leads to changes in metabolites that affect efficacy. The processing of CMM is called *paozhi*, an indispensable production process. The traditional processing methods are based primarily on the theory and the character of CMM which includes various methods, like parching, roasting, moistening, burning. The purpose of processing is eliminating or reducing the toxicity of drugs, strengthening the curative effect, facilitating the preparation or storage, and making the drugs pure.

#### Metabolite change detection

Changes in drug composition caused by processing will alter their clinical effects. UPLC-QTOF-MS based metabolomic paired with novel informatics UNIFI platform have been undertaken for evaluating the changes in chemical components of *Astragali Radix* after processing. PCA scores and OPLS-DAS maps indicate that there are some changes in compound composition of *Astragali Radix* processed by heating and auxiliary materials. For example, the content of Calycosin-7-O-β-Dglucopyranoside varies significantly after processing, while several unknown compounds are newly generated after processing, which can applied as the biomarkers. The present study provided a basis of chemical components for revealing connotation of different processing techniques on *Astragali Radix* [80]. Similarly, different processed and raw products of *Radix Polygala* were studied by NMR and UPLC. PCA analysis results showed that the metabolic composition of *Radix Polygala* was obviously different. Moreover, there are also differences in metabolomics between *Radix Polygala* honey products and licorice products [81]. Ginsenoside, a biomarker of raw and steamed *Panax notoginseng*, also was discoveried by UPLC/TOF–MS [82]. Through plant metabolomics, changes in metabolites of CMM before and after processing can be more intuitively found, and the relationship between processing and efficacy can be more directly clarified if combined with corresponding pharmacodynamic studies. The purpose of processing toxic drugs is often to reduce toxicity and increase efficiency, which can also be tested by plant metabolomics. Pre-use process is necessary due to long-term usage of *Rheum palmatum* may bring liver and kidney injury. In one study, UPLC/Q-TOF–MS based metabolomic was applied to study differential chemical composition of *R. palmatum* samples before and after processing. The results showed that Emodin-8-O-glucoside and gallic acid-3-O-glucoside can distinguish raw and processed products as biomarkers [83]. The detoxification effects of processing of Fuzi (the lateral root of *Aconitum carmichaelii Debx*) were evaluated via RPLC-Q-TOF/MS coupled with PCA, which found 19 key biomarkers associated with detoxification [84]. In addition, an UPLC-MS method was established to analyze the chemical composition before and after the processing of *Euphorbia pekinensis* root. The change of chemical composition caused by processing is obviously altered, which is supposed to be the material basis of the detoxification effect [85]. The reduction of toxic for CMM is an important target for processing, and mining attenuated related metabolites of toxic CMM with metabolomics provides criteria for clinical drug safety.

#### Processing methods evaluation

The evaluation of processing methods is advantageous to standardize the production process and excavate suitable and efficient processing methods of CMM. The processing method of ginseng is mainly steaming. Chan et al. utilized a UHPLC/TOF–MS based metabolomic strategy to discriminate the difference of steaming *Panax notoginseng* [86]. Moreover, Kim et al. compared the metabolite changes of steamed *ginseng* at 100 °C, 110 °C and 120 °C, and found that 120 °C is more conducive to its efficacy. At the same time, it was also noted that steaming caused the formation of new ginsenoside [87]. The quality of the drying method will affect the content of the active compounds in CMM. Chromatography-mass spectrometry coupled with targeted and untargeted analyses was applied to study on the relationship between drying method and chemical concentration of *Notopterygium franchetii*. 30 differentiated compounds are detected in seven drying methods. The results suggested that hot air drying is the best processing method, with minimal chemical changes at the lowest cost, and the largest chemical change caused by shadow drying [88]. Dai et al. used NMR and LC-DAD-MS method to study the effect of stress caused by water loss on metabolites in root of *Salvia miltiorrhiza*. Using freeze drying as reference, the effects of sun drying and air drying methods were compared. The results showed that water stress resulted in a significant change in the profile of metabolites in *salvia miltiorrhiza*, and content of tanshinone was significantly increased by air-drying [89]. Plant metabolomics has been widely used to evaluat the drying methods of CMM, but less research in other processing methods, which is also the need to be expanded later, such as stir-frying with bran, stir-frying with honey, steaming with wine and so on. Furthermore, evaluation of the postharvest peeling process of *Astragalus* root using 1HNMR and UPLC-MS has been demonstrated that there was a clear distinction between exfoliated and undelayed root of *astragalus*. Peeling can cause significant loss of several primary metabolites, while the content of xylem-related compounds were higher [90]. Postharvest processing often determines the quality of herbal medicines and rhizomes plants, but an full analysis using several complementary methods has not been developed. Plant metabolomics coupled with chemometric analysis can be applied to ensure proper postharvest processing by offering information of primary and secondary metabolites.

### Herbal marketing

In drug sales, adulteration, staining, excessive sulfur fumigation will affect the changes of small molecular compounds in CMM, and finally reflect the difference of product quality. Plant metabolomics can be used to detect the indexes and evaluate the quality problems in the sales of CMM.

**Fig. 4:**
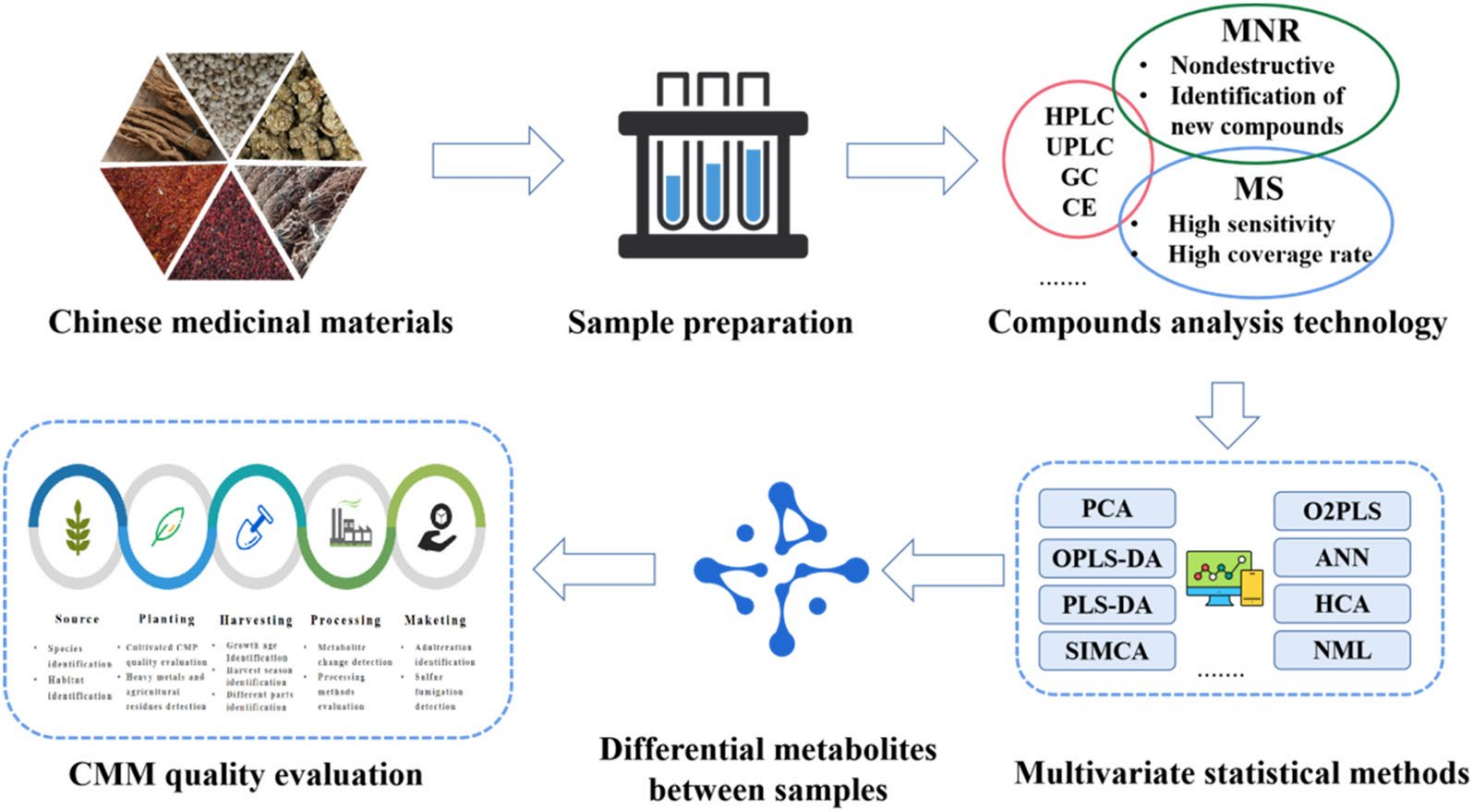
Schematic diagram of experimental steps of plant metabolomic in quality evaluation of CMM

In recent years, UPLC-MS based metabolomics were applied to analysis 35 commercial products of *Hydrastis canadensis*. The obtained results demonstrate the potential for untargeted metabolomics to discriminate between multiple unknown products and predict possible adulteration [91]. While *Panax ginseng* adulteration is rampant, Nguyen et al. used 1H NMR combined with OPLS-DA to distinguish 60 *Panax ginseng* samples from Korea and China. 21 mixed samples of numerous Korea/China ratios were tested, showing satisfactory separation according to the proportion of mixing [92]. It is noteworthy that sulfur fumigation bleaching is also a common adulteration method of ginseng in the market which requires strict control. UPLC-QTOF-MS/MS-based metabolomics approach was developed to evaluate the holistic qualities of commercial white ginseng (WG) and red ginseng (RG) from herbal markets. 43 compounds including 3 sulfur-containing compounds were identified within 24 min suggested the inconsistencies in the quality of commercial WG and RG due to heat treatment and sulfur fumigation [93]. UPLC-ESI-QTOF-MS/MS-based non-targeted metabolomics and UPLC-ESI-QTRAP-MS/MS-based widely targeted metabolomics identified five characteristic sulfur fumigation markers of *Trichosanthis Radix* (TR) samples. At the same time, the different sulfur fumigation degree of TR samples was tested by chemical transformation analysis and sulfur dioxide residue test. Furthermore, 20 non-sulfur labeled metabolites were tested to evaluate the quality of samples of TR before and after sulfur fumigation. Sulfur fumigation of CMM may cause harm to human body or affect clinical effects and needs to be strictly controlled. The plant metabolome has provided technical support for the detection of sulfur-fumigated drugs by providing information about sulfur fumigation markers [94]. The scarcity of wild *Rhodiola officinalis* resources, coupled with strong market demand, led to the emergence of artificial cultivation of low quality varieties as adulterated drugs, seriously affected the CMM market. Booker et al. combined with NMR and high performance thin layer chromatography (HPTLC) technology to study the chemical composition differences of five varieties of *Rhodiola officinalis* in the market, proved that salidroside and tyrosine were the biomarkers, and established the method of content identification in mixed components to provide a methodological basis for distinguishing adulterated medicinal materials [95]. Metabolomics approaches have been applied to evaluate the quality and quantity of characteristic molecules in three different *Rhodiola* species, *Rhodiola rosea*, *Rhodiola kirilowii* and *Rhodiola crenulata.* Main molecules were identified by one and two-dimensional NMR metabolomics and quantified by HPLC–UV assay, and then OPLS-DA reveals specific patterns in the metabolite profiles. Therefore, plant metabolomics can be utilized to study the chemical diversity of different species, identify unique metabolites and identify adulterated products [96]. Degree of *Angelica acutiloba* root was evaluated by GC TOF–MS combined with PCA as the good, the moderate and the bad. Among the 22 metabolites identified, phosphoric acid, proline, malic acid, and citric acid showed an association with high-quality roots, while fructose and glucose are mostly found in bad roots. Moreover, PCA shows that malic acid is the most differentiated for medium quality roots [97]. The market price of different levels of CMM varies greatly. The adulteration of CMM occurs repeatedly due to the evaluation methods of different levels are not unified. Plant metabolomics can be applied as a evaluation methodology to provide the theoretical support for sales based on quality (Fig. 4).

## Concluding and future remarks

CMM is integrity due to complex composition, and various components interact to produce unique efficacy. Each link of CMM production may cause changes in metabolites and affect the efficacy. A quality control system to respectively evaluate production link from source, planting, harvesting, processing and marketing is urgently needed. Plant metabolomics technique can be used to analyze the composition and variation of metabolites from the view of the whole, which coincides with the “holistic” and “dynamic” views of CMM. In recent year, plant metabolomics have been applied for metabolites analysis to identify species, habitats, growth age, harvest season, parts and adulteration of CMM, which show an enormously potential for quality evaluation [98]. Although plant metabolomics develops rapidly, it is still in the early stage of development. There are still some limitations of plant metabolomics due to the diversity and complication of metabolites, and performance flaw of the instruments [99, 100]. First, the metabolites of CMM are complex and are vulnerable to impact by many factors, including heredity, temperature, humidity, light, chemical substances, etc., which poses challenges to metabolomic analysis [101]. Second, the application conditions of numerous technologies have limitations, where there is not an untargeted approach can profile all the metabolites of CMM. Third, the existing databases are not perfect because many studies on metabolism of medicinal plants remain blank, which limits the analysis and identification of chemical components. Therefore, breaking technical barriers and building more complete databases for comprehensively profiling metabolites are the primary problem of plant metabolomics. Systematically excavating and processing mass data is still the technical bottleneck of current plant metabolomics, which need a high coverage data acquisition strategy to min information of low abundance metabolites. Moreover, establishing a new approach for sample acquisition and preparation is beneficial to improve the experimental reproducibility and stability.

**Fig. 5:**
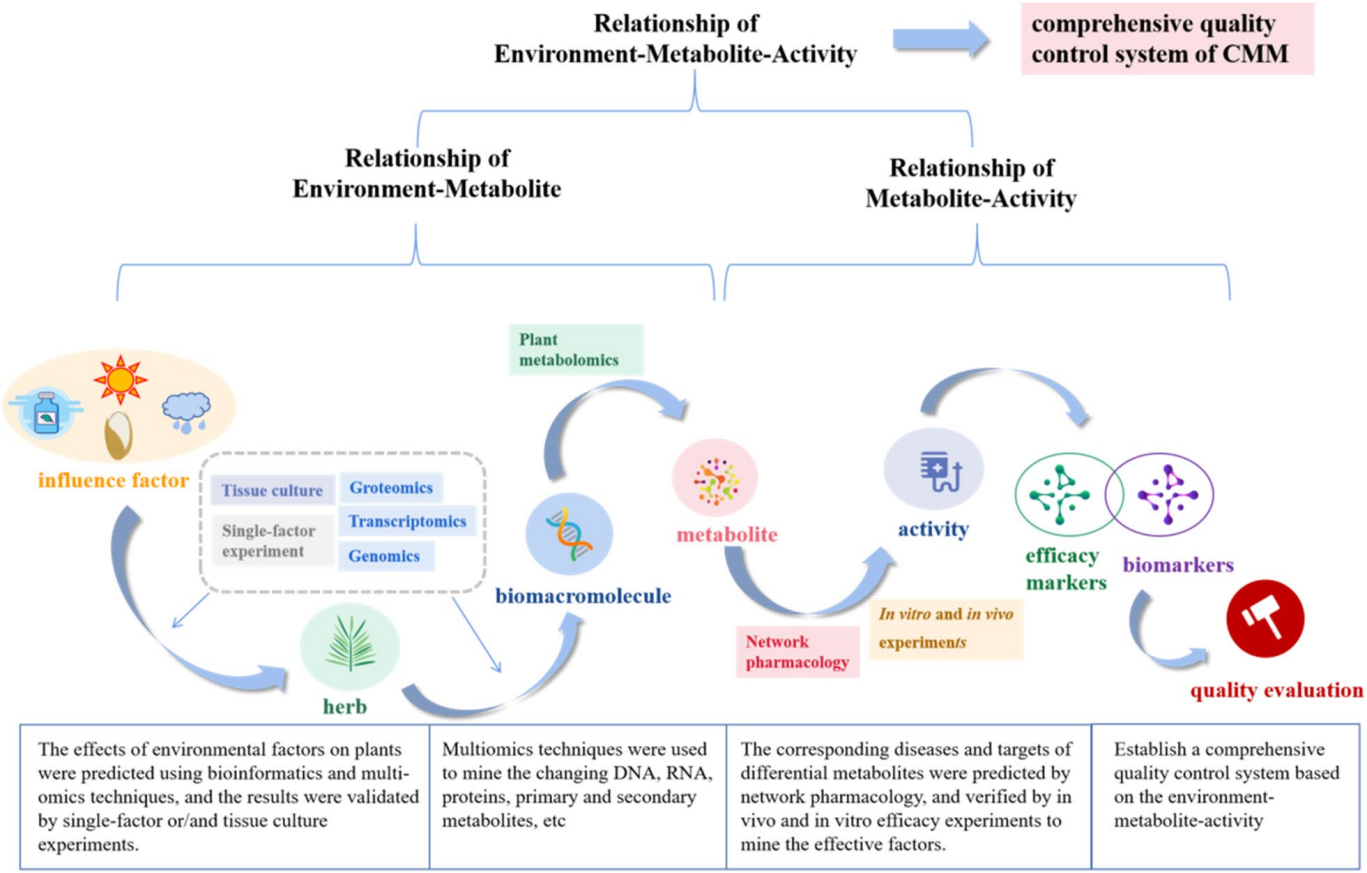
Schematic diagram of multi-disciplinary application based on plant metabolomics to establish a comprehensive CMM quality control system

The application of plant metabolomics in the field of quality control will have more development space with the improvement of new systems and multidisciplinary integration applications. Establishing a comprehensive quality control system needs to improve the relationship of environment-metabolites and metabolites-activity. Combining multi-omics technology and bioinformatics technology, the influence of external factors on plant metabolism can be explored from the gene level and a comprehensive metabolic network can be established [102–105]. Based on the differential metabolites found by plant metabolomics, the network pharmacology can be used to predict the disease and target, and finally discover the therapeutic material basis and the predict the active mechanism of CMM. The relationship between different metabolites and their activity can be clarified by plant metabolomics combined with in vivo and in vitro efficacy experiments, then active markers of CMM can be discovered [106–110]. Ultimately, an associative network between environment-metabolite-efficacy will be established for a standardized cultivation-harvesting-processing system and a quality evaluation method of CMM. This system will substantially promote the production and application of CMM towards scientification and modernization. (Fig. 5).

## Data Availability

Not applicable.

## Abbreviations

CMM: Chinese Medicinal materials
ChP: Chinese pharmacopoeia
NMR: Nuclear magnetic resonance
MS: Mass spectrometry
LC: Liquid chromatography
GC: Gas chromatography
CE: Capillary electrophoresis
HPLC: High performance liquid chromatography
UPLC: Ultra performance liquid chromatography
UV: Ultraviolet spectrum
IR: Infrared
EI: Electron impact ion source
CI: Chemical ionization
ESI: Electrospray ionization
APCI: Atmospheric pressure chemical ionization
MALDI: Matrix-assisted laser desorption ionization
DESI: Desorption electrospray ionization
Q: Quadrupole
IT: Ion trap
QQQ: Triple quadrupole
TOF: Time-of-flight
FTICR: Fourier transform ion cyclotron resonance
HRMAS: 1H high resolution magic angle rotation
PCA: Principal components analysis
PLS-DA: Partial least squares discriminative analysis
OPLS-DA: Orthogonal projections to latent structures
DART: Direct Analysis in Real Time
KWG: *Korean* *white* *ginseng*
CWG: *Chinese* *white* *ginseng*
RRLC: Rapid resolution liquid chromatography
ICP-AES: Plasma atomic emission spectro
ICP-MS: Inductively coupled plasma-mass spectrometry
FTIR: Fourier Transform infrared spectroscopy
ICD: Identification, classification and differentiation
PCR: *Pericarpium Citri Reticulatae*
PCRV: *Pericarpium Citri Reticulatae Viride*
HELP: Heuristic evolving latent projection
WG: White ginseng
RG: Red ginseng
TR: *T* *richosanthis Radix*
HPTLC: High performance thin layer chromatography
Q-markers: Quality marker
